# Two New Meal- and Web-Based Interactive Food Frequency Questionnaires: Validation of Energy and Macronutrient Intake

**DOI:** 10.2196/jmir.2458

**Published:** 2013-06-05

**Authors:** Sara E Christensen, Elisabeth Möller, Stephanie E Bonn, Alexander Ploner, Antony Wright, Arvid Sjölander, Olle Bälter, Lauren Lissner, Katarina Bälter

**Affiliations:** ^1^Karolinska InstitutetDepartment of Medical Epidemiology and BiostatisticsStockholmSweden; ^2^UK Medical Research CouncilMRC Collaborative Center for Human Nutrition ResearchCambridgeUnited Kingdom; ^3^KTH - Royal Institute of TechnologySchool of Computer Science and CommunicationStockholmSweden; ^4^University of GothenburgDepartment of Public Health and Community MedicineGothenburgSweden

**Keywords:** validity, reproducibility, food frequency questionnaire, Internet, weighed food record, doubly labeled water, adult

## Abstract

**Background:**

Meal-Q and its shorter version, MiniMeal-Q, are 2 new Web-based food frequency questionnaires. Their meal-based and interactive format was designed to promote ease of use and to minimize answering time, desirable improvements in large epidemiological studies.

**Objective:**

We evaluated the validity of energy and macronutrient intake assessed with Meal-Q and MiniMeal-Q as well as the reproducibility of Meal-Q.

**Methods:**

Healthy volunteers aged 20-63 years recruited from Stockholm County filled out the 174-item Meal-Q. The questionnaire was compared to 7-day weighed food records (WFR; n=163), for energy and macronutrient intake, and to doubly labeled water (DLW; n=39), for total energy expenditure. In addition, the 126-item MiniMeal-Q was evaluated in a simulated validation using truncated Meal-Q data. We also assessed the answering time and ease of use of both questionnaires.

**Results:**

Bland-Altman plots showed a varying bias within the intake range for all validity comparisons. Cross-classification of quartiles placed 70%-86% in the same/adjacent quartile with WFR and 77% with DLW. Deattenuated and energy-adjusted Pearson correlation coefficients with the WFR ranged from *r*=0.33-0.74 for macronutrients and was *r*=0.18 for energy. Correlations with DLW were *r*=0.42 for Meal-Q and *r*=0.38 for MiniMeal-Q. Intraclass correlations for Meal-Q ranged from *r*=0.57-0.90. Median answering time was 17 minutes for Meal-Q and 7 minutes for MiniMeal-Q, and participants rated both questionnaires as easy to use.

**Conclusions:**

Meal-Q and MiniMeal-Q are easy to use and have short answering times. The ranking agreement is good for most of the nutrients for both questionnaires and Meal-Q shows fair reproducibility.

## Introduction

The food frequency questionnaire (FFQ) is a commonly used method for assessing diet in large-scale epidemiological studies. The advantages of the FFQ include a low participant burden compared to dietary records, and low cost because it is typically a self-administered method. However, there is a need for methodological improvement, including the FFQ layout and its ease of use.

Most FFQs list food items according to food groups (vegetables, meats, dairy, etc), yet people typically consume food grouped into meals. Moreover, meal-based questionnaire designs have been shown to facilitate recall of dietary intake in previous studies [1,2]. Therefore, we developed a meal- and Web-based FFQ, called Meal-Q, with a design that allows for individually adapted follow-up questions. Thus, participants only answer questions relevant to their own food habits. For example, a high consumer of bread and cheese will get follow-up questions about the number of slices of bread and cheese, whereas a low consumer will not. This feature reduces the answering time and improves the ease of use.

Approximately 90% of the Swedish adult population used the Internet in 2011 [3], justifying development of Web-based questionnaires for national population-based studies. Furthermore, the Web-based design makes the use of Meal-Q more cost-efficient than a paper-based FFQ and facilitates assessment of large samples. The ability to use built-in checks for missing answers and the immediate transfer of answers into digital format also assures complete data collection and improves data quality [4,5].

We evaluated the validity and reproducibility of energy and macronutrient intake assessed with Meal-Q by comparing it to a weighed food record (WFR) and doubly labeled water (DLW). By using truncated data from Meal-Q, we also validated a shorter version called MiniMeal-Q.

## Methods

### Background

The development of Meal-Q was based on results from a population-based study in which 700 randomly selected Swedish participants reported, through either face-to-face interviews or telephone 24-hour recalls, on the food products they consumed for breakfast, lunch, dinner, and snacks (E Möller and S Christensen, personal written communication, August 2008). This dietary information guided the design of a meal- and Web-based FFQ called MaxMeal-Q. After a pretest of MaxMeal-Q in a randomly selected group of individuals (N=216), the shorter version, Meal-Q, was formed by omitting less commonly consumed food items and dishes. Subsequently, Meal-Q was included in the Validation of Methods Assessing diet and physical activity (VALMA) study. The reference methods were a 7-day WFR on the Web and DLW for estimation of energy expenditure [6].The Research Ethics committee at Karolinska Institutet approved the study.

After the validation study was completed, researchers from LifeGene, a large population-based cohort study [7], decided to use Meal-Q under the condition that the answering time be reduced. Therefore, we developed the shorter version, MiniMeal-Q, by omitting food items consumed on average with a low intake frequency and that contributed least to the total energy and nutrient intake. Yet, food items representing important food sources of certain nutrients were kept (eg, black pudding that contributes to iron intake). After a time test, LifeGene decided to use MiniMeal-Q. We validated MiniMeal-Q in the present study by truncating Meal-Q data to simulate MiniMeal-Q. The inherent dependence between Meal-Q and MiniMeal-Q should be taken into account when comparing their validity.

### Recruitment

In April 2009, 180 healthy volunteer men and women aged 20 to 63 years were recruited to the VALMA study through public advertisement in Stockholm County, Sweden. Recruitment also took place at 3 universities including announcements among nutritionist students. Access to the Internet and an email address were prerequisites for eligibility, as well as not being on a weight-loss diet, not being pregnant, and not having given birth within the past 10 months. At an introductory meeting, participants were informed about the study and signed informed consent forms. Participants self-reported their height and weight, which was used to calculate body mass index (BMI).

### Study Design

**Figure 1:**
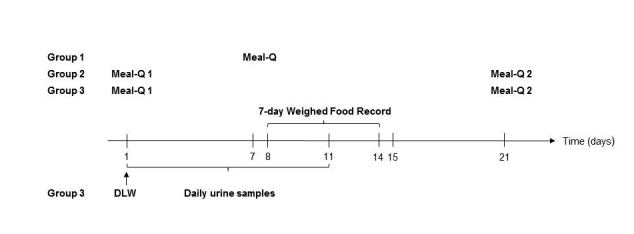
The 3-week study scheme of the VALMA study.

After recruitment, the participants were divided into 3 age- and gender-balanced groups: group 1 (n=87), group 2 (n=53), and group 3 (n=40). Each group followed a 3-week study scheme shown in Figure 1.

All groups filled out Meal-Q and the WFR on the Web at their own choice of location (eg, at home) and group 3 was also given DLW. Groups 2 and 3 filled out a second Meal-Q after 3 weeks. Data from the first administered Meal-Q assessment and the WFR from all groups were compared for validity evaluation. The data from the first Meal-Q assessment was truncated for simulated analysis of MiniMeal-Q. The first and second Meal-Q assessments from groups 2 and 3 were compared for reproducibility evaluation. Information about education, occupation, and tobacco use (smoking and Swedish snuff) was collected in the first questionnaire. Answering time was automatically recorded, and directly after completion of the first Meal-Q, a short Web survey followed to evaluate its ease of use.

### Dietary Assessment

#### Meal-Q

**Figure 2:**
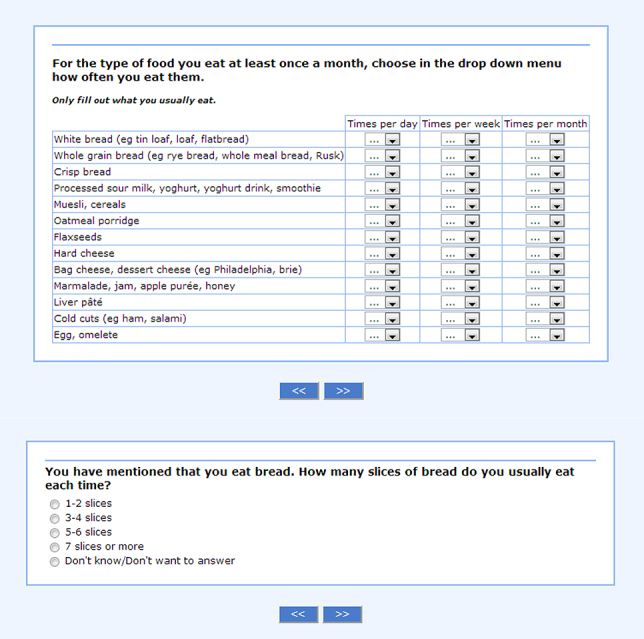
Screenshot of a Meal-Q module: breakfast and snack items and follow-up question on bread (translated from the Swedish questionnaire version in the VALMA study).

The interactive Meal-Q included 102 to 174 food items (depending on the number of follow-up questions) and asked about dietary intake during the past few months. For an example of a questionnaire module, see Figure 2. Meal-Q assessed intake of (1) food items, dishes, and beverages, (2) energy and nutrients, including alcohol, (3) supplements, (4) meal patterns, and (5) eating behavior, such as restaurant visits, intake of fast food, light products, probiotics, and the use of cooking fat and salt. Respondents chose from predefined food items and intake frequencies and only filled in what they ate at least once a month. For each of the following food groups, 5 photos of portion sizes were included: (1) rice, potatoes, and pasta, (2) meat, chicken, fish, and vegetarian substitutes, and (3) vegetables (raw or cooked). The photos were used to calculate portion sizes for cooked dishes and vegetables. For other food items, standard portion sizes were used based on information from the National Food Agency, the Swedish Consumer Agency, measured portion sizes developed by the research group, as well as standard portion sizes used in other FFQs at Karolinska Institutet.

#### MiniMeal-Q

MiniMeal-Q includes 75 to 126 food items—approximately 30% fewer items than Meal-Q—and has similar questions on meal patterns and eating behavior. After the VALMA study was finished, MiniMeal-Q was sent out to 79 volunteer VALMA participants to assess answering time and ease of use.

#### Weighed Food Records on the Web

At study start, participants were given oral instructions, a kitchen scale, and a handbook with instructions on how to complete the 7-day WFR by using a Web-based program. Participants were asked to weigh and report all consumed food products and beverages at the highest detail level possible (eg, each food item in a dish was encouraged to be reported in its individual weight). The participants could choose among over 2000 food items in the program’s food database, and they also recorded which day they consumed the food item as well as for which meal (ie, breakfast, lunch, dinner, or between meals). Data collectors checked all records for completeness. In the program, participants also provided a 7-day pedometer-based physical activity record. The participants were asked to report their total number of daily steps as well as other activities not captured by pedometers, such as bicycling or swimming. From this, the physical activity level (PAL) was calculated for each participant and the information was used for identification of potential energy underreporters in the WFR by using the Goldberg cut-off method [8].

#### Nutrient Database

Intake of food items and dishes from Meal-Q, MiniMeal-Q, and the WFR were converted into energy (kJ/day) and macronutrient (g/day) intake using the national database on nutrient content published by the Swedish National Food Agency [9]. The nutrient conversion for the questionnaires was done by computer programs developed and validated specifically for this study, whereas the conversion of the WFR was done with the Web-based WFR program. Dietary supplements were not included in the analyses.

### Doubly Labeled Water Method

Total energy expenditure was determined in group 3 (n=40) using the DLW method [10] over 11 consecutive days (Figure 1). The details of this procedure have been described previously [11]. Briefly, on day 1 at the study site, each participant provided a 5-milliliter urine sample before receiving an oral DLW dose calculated according to body weight [12]. Subsequently, daily spot urine samples were collected for a total of 9 days. Participants were instructed to collect urine samples at a similar time each day (but not the first void of the day). All samples were kept refrigerated. On day 11, the 9 urine samples were returned to the study site and the eleventh urine sample was collected. All samples were shipped to the Medical Research Council, Human Nutrition Research, Cambridge, United Kingdom, for isotopic analysis, which has been previously described in detail [13]. Enrichments of ^2^H/^1^H and ^18^O/^16^O in urine samples were determined by mass spectrometry. Following conversion to the universally accepted Vienna Standard Mean Ocean Water (VSMOW) / Standard Light Arctic Precipitation (SLAP) scale, total energy expenditure (TEE) was calculated by using standard equations [14-16]. CO_2_ production (mole/day) was estimated using Schoeller et al’s correction for fractionation [15] and a respiratory quotient of 0.85. The respiratory quotient is based on omnivores with 30% to 35% energy contribution from fat and suitable to the VALMA population. The results of the CO_2_ production were used to calculate the TEE of each participant by using the modified Weir equation [17].

### Statistical Analysis

Descriptive characteristics of study participants are presented as mean (SD) and as counts (%). Differences in BMI and age between study groups, between men and women, and between included and excluded participants were assessed using a 2-sample *t* test. Differences in education, nutrition background (studying or working in the nutrition field), and tobacco use were assessed using Fisher’s exact test. The level of statistical significance was set to alpha =.05.

Median and interquartile range (IQR) of crude energy and macronutrient intake was calculated and compared among Meal-Q, MiniMeal-Q, and the WFR. Energy intake from the questionnaires was also compared to TEE from DLW. Wilcoxon signed rank tests were used to determine differences between all methods. The median (IQR) answering time in minutes of each questionnaire was calculated and ease of use was evaluated from the Web survey. The between-person variance captured in the truncated MiniMeal-Q as compared to Meal-Q was calculated using linear regression.

For validity and reproducibility analyses, macronutrients were adjusted for total energy intake using the residual method [18]. Variables deviating from the normal distribution were transformed using the square, square root, or log transformation, as appropriate. Absolute agreement and potential difference in bias within the intake range were evaluated by plotting the differences between questionnaires and WFR or DLW against the average of the 2 methods, according to the method of Bland and Altman [19]. The degree of variation was represented by the limits of agreement, ie, ±2 SD of the mean difference. The ranking agreement and magnitude of misclassification when comparing questionnaires with the WFR and DLW was tested by dividing participants into quartile categories of energy and energy-adjusted macronutrient intake. Proportions of participants classified into the same, adjacent, and extreme quartiles were calculated. Because variables were normally distributed after energy adjustment and transformation, Pearson correlation coefficients were used to measure the degree of linear relationship between the questionnaires and the WFR and DLW. Deattenuated correlations corrected for within-person variation in the WFR were calculated using the formulas of Beaton et al [20] and Liu et al [21], and confidence intervals (CI) were produced using the method of Willett and Rosner [22]. Confidence intervals for correlations with DLW were obtained using the bootstrap method [23].

Reproducibility of Meal-Q was evaluated by comparing crude median energy and macronutrient intake between the first and second Meal-Q and by cross-classification of energy and energy-adjusted [18] quartiles of macronutrient intake. Intraclass correlation coefficients (ICCs) [24] were also computed using 1-way ANOVA with random effects. Statistical analyses were performed using STATA statistical software version 11.2 (StataCorp LP, College Station, TX, USA).

## Results

One participant was excluded due to dropout (group 1) and 2 others due to illness (group 2 and 3). Eleven participants (4 in group 1, 6 in group 2, and 1 in group 3) were identified as energy underreporters by applying the Goldberg cut-off [8] on energy intake from the WFR together with data from each participant’s calculated PAL. Additionally, 3 underreporters (group 3) in the WFR were identified using individual PAL values calculated from DLW data. Because of the implausible energy intake by the WFR, the 14 underreporters were excluded for the validity comparison between Meal-Q, MiniMeal-Q, and the WFR; therefore, 163 participants remained (group 1: n=82; group 2: n=46; and group 3: n=35). For the validity comparison between Meal-Q, MiniMeal-Q, and DLW in group 3, no exclusion of energy underreporters was made; therefore, 39 participants remained. For the reproducibility analysis of Meal-Q, 4 participants had missing values in the second administered Meal-Q; therefore, 87 participants remained. We found no statistically significant differences between included and excluded participants in terms of age, BMI, education, nutrition background, or tobacco use.

### Descriptive Statistics

As shown in Table 1, most of the study participants were students or highly educated. One-third were working full time, and almost as many had a nutrition background. Few participants used tobacco. There was no statistically significant difference between groups or sexes regarding age, BMI, education, nutrition background, or smoking (but more men than women used Swedish snuff).

**Table 1:** Characteristics of the participants in the validation study (n=167^a^).

Characteristics		By group			By gender		All (n=167)
		Group 1 (n=82)	Group 2 (n=46)	Group 3 (n=39)	Men (n=35)	Women (n=132)	
**Gender, n (%)**							
	Male	16 (19.5)	11 (23.9)	8 (20.5)			35 (21.0)
	Female	66 (80.5)	35 (76.1)	31 (79.5)			132 (79.0)
Age (years), mean (SD)		34 (12)	31 (11)	33 (12)	33 (10)	33 (12)	33 (12)
BMI (kg/m^2^), mean (SD)		23 (3.6)	23 (3.4)	23 (3.7)	24 (2.2)	23 (3.8)	23 (3.6)
Education >12 years, n (%)		64 (78.0)	38 (82.6)	32 (82.1)	28 (80.0)	106 (80.3)	134 (80.2)
Working full time, n (%)		33 (40.2)	12 (26.1)	10 (25.6)	12 (34.3)	43 (32.6)	55 (32.9)
Student, n (%)		41 (50.0)	31 (67.4)	26 (66.7)	19 (54.3)	79 (59.8)	98 (58.7)
Background in nutrition^b^, n (%)		21 (25.6)	15 (32.6)	13 (33.3)	6 (17.1)	43 (32.6)	49 (29.3)
Tobacco use^c^, n (%)		11 (13.4)	5 (10.9)	6 (15.4)	12 (34.3)	10 (7.6)	22 (13.2)

^a^From this study sample, 4 underreporters were excluded for analysis with the WFR (n=163). There were no statistically significant differences in characteristics between groups or sexes, except for Swedish snuff between sexes (1.8% women and 4.2% men, *P*=.001) via 2-sample *t* test and Fisher’s exact test.

^b^Studying or working in the nutrition field.

^c^Tobacco use = smoking and/or Swedish snuff. Values are missing for 3 women in group 3.

The median time to answer the Meal-Q and the MiniMeal-Q was 17 (IQR 11) and 7 (IQR 4) minutes, respectively. Most (92%) participants perceived Meal-Q as easy to fill out, 91% thought the questions were relevant, and 93% reported that food items and dishes were presented in a logical order. For MiniMeal-Q, the figures were 95%, 88%, and 91%, respectively. The overall mean grade of Meal-Q and MiniMeal-Q’s ease of use was 4.2 on a 5-point scale in which 5 was the best grade. The between-person variance captured by MiniMeal-Q as compared to Meal-Q ranged from 96% to 99% for energy and macronutrients.

### Validity

Energy and macronutrient intake was higher in the WFR compared with both questionnaires, except for polyunsaturated fat assessed with Meal-Q (Table 2). In group 3 (n=39), the energy expenditure from DLW was higher than energy intake assessed by both questionnaires (*P*<.001, Wilcoxon’s signed rank test). The energy expenditure from DLW was 11,423 kJ (IQR 2777) and the energy intake assessed with Meal-Q and MiniMeal-Q were 7954 kJ (IQR 2736) and 7358 kJ (IQR 2718), respectively.

**Figure 3:**
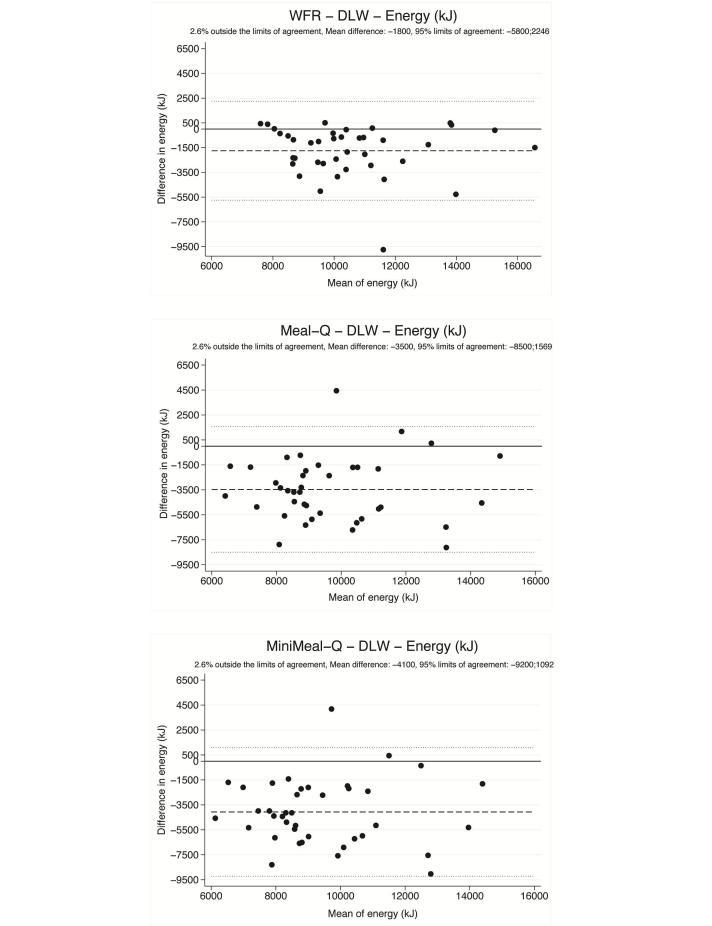
Bland-Altman plots showing the differences in energy intake assessed with the WFR, Meal-Q, and MiniMeal-Q and the energy expenditure measured with DLW plotted against the mean of the 2 methods (n=39). Each data point represents 1 participant. The long-dashed line shows the mean difference. The short-dashed lines show the 95% limits of agreement (mean difference ±2 SD).

As shown in Figure 3, the Bland-Altman plots with DLW indicate that the WFR and both questionnaires underestimated energy intake for most participants. Compared to the WFR, the questionnaires had a larger underestimation, larger variance, and a weak trend of decreasing accuracy with increasing intakes.

**Figure 4:**
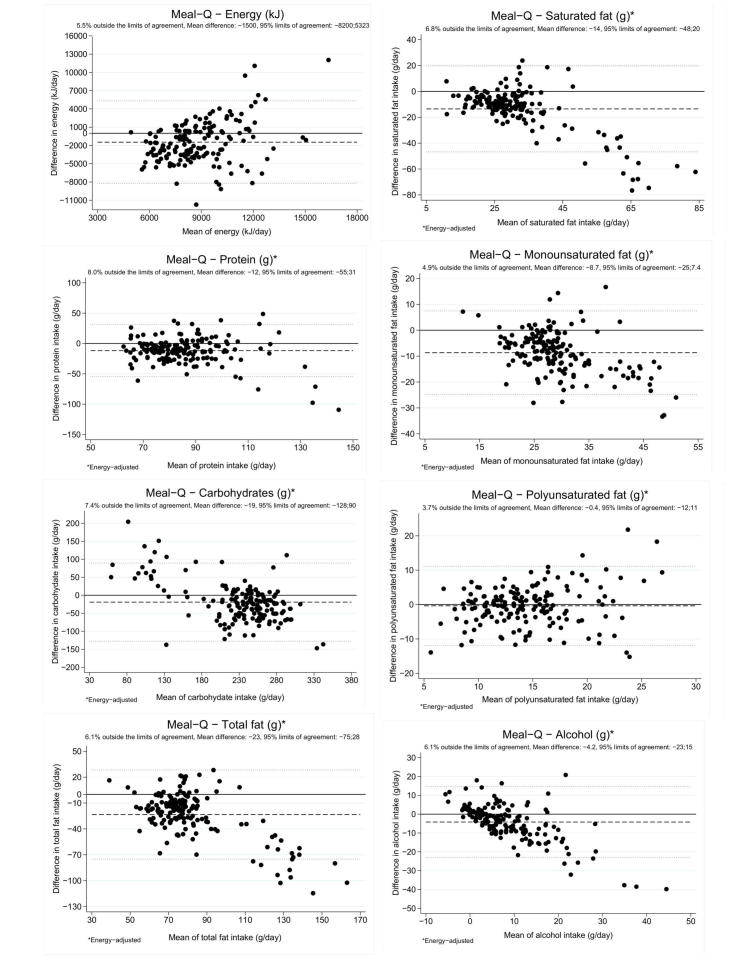
Bland-Altman plots showing the differences in energy, protein, carbohydrate, total fat, saturated fat, monounsaturated fat, polyunsaturated fat, and alcohol intake assessed with Meal-Q and intake assessed with the WFR plotted against the mean of the 2 methods (N=163). Macronutrients are energy-adjusted using the residual method [18].

The Bland-Altman plots of Meal-Q and the WFR in Figure 4 showed a negative mean difference for energy and all macronutrients. There was a trend of decreasing accuracy with increasing energy and polyunsaturated fat intake, and trends of increasing underestimation with increasing intakes for other macronutrients. Because of varying bias within the intake range, the proportion of participants outside the limits of agreement deviated somewhat from 5% for some plots. Bland-Altman plots for MiniMeal-Q and the WFR were very similar to those for Meal-Q and the WFR (see Multimedia Appendix 1).

Table 3 shows that the proportion of participants classified into the same or adjacent quartile for energy was 70% by Meal-Q and 67% by MiniMeal-Q as compared to the WFR. Correspondingly, the proportions for macronutrients ranged from 76% to 86% for both questionnaires. Quartile cross-classification of Meal-Q and DLW placed 77% into the same or adjacent quartile, and values were identical for MiniMeal-Q.

Pearson correlation coefficients (*r*) with the WFR and DLW were similar between Meal-Q and MiniMeal-Q (Table 4). Deattenuated and energy-adjusted correlations with the WFR ranged from *r*=0.18-0.73 for Meal-Q and from *r*=0.18-0.74 for MiniMeal-Q. Correlation with DLW was *r*=0.42 for Meal-Q and *r*=0.38 for MiniMeal-Q.

#### Reproducibility

Table 5 shows that there were no statistically significant differences in crude intakes between the first and second Meal-Q assessments. The proportion of participants classified into the same or adjacent quartile ranged from 85% to 96%. ICCs ranged from *r*=0.43-0.92 for crude intakes and from *r*=0.57-0.90 for energy-adjusted macronutrients.

**Table 2:** Daily crude energy and macronutrient intake assessed with the WFR, Meal-Q, and MiniMeal-Q (n=163).

Energy and macronutrients	WFR	Meal-Q^a^		MiniMeal-Q^a^	
	Median (IQR)	Median (IQR)	% of WFR	Median (IQR)	% of WFR
Energy (kJ)	9183 (2340)	7667 (3723)	83	7017 (3632)	76
Protein (g)	85 (37)	79 (40)	93	70 (34)	82
Carbohydrates (g)	243 (97)	211 (132)	87	190 (124)	78
Total fat (g)	86 (37)	65 (34)	76	62 (35)	72
Saturated fat (g)	33 (18)	22 (14)	67	20 (13)	61
Monounsaturated fat (g)	31 (16)	23 (13)	74	22 (11)	71
Polyunsaturated fat (g)	14 (8)	13 (8)	93	12 (9)	86
Alcohol (g)	6 (15)	5 (8)	85	5 (8)	83

^a^Intakes assessed with Meal-Q and MiniMeal-Q were statistically significantly different from the WFR (*P*=.01), except for polyunsaturated fat assessed with Meal-Q (*P*=.28). Intakes assessed with Meal-Q and MiniMeal-Q were statistically significantly different from each other (*P*<.001) via Wilcoxon signed rank test.

**Table 3:** Quartile cross-classification of mean daily energy and energy-adjusted^a^ macronutrient intake assessed with Meal-Q, MiniMeal-Q, and the WFR (n=136) and cross-classification of mean daily energy intake and energy expenditure measured with DLW (n=39).

Energy and macronutrients	Same quartile, %		Adjacent quartile, %		Extreme quartile, %	
	Meal-Q	MiniMeal-Q	Meal-Q	MiniMeal-Q	Meal-Q	MiniMeal-Q
Energy	26	27	44	40	8.5	7.4
Protein	36	40	40	36	6.7	5.5
Carbohydrate	42	42	40	37	2.5	1.8
Total fat	37	33	41	46	8.0	9.2
Saturated fat	52	45	33	37	4.9	4.3
Monounsaturated fat	44	44	33	33	6.1	6.7
Polyunsaturated fat	33	31	47	49	5.5	4.9
Alcohol	50	49	36	37	4.3	3.7
DLW, energy (kJ)	33	33	44	44	2.6	2.6

^a^Adjustments for total energy intake were made using the residual method [18].

**Table 4:** Pearson correlation coefficients between Meal-Q, MiniMeal-Q, and the WFR (n=163) and DLW (n=39).

Energy and macronutrients	Crude^a^		Energy-adjusted^a,b^		Deattenuated (95% CI)^c^	
	Meal-Q	MiniMeal-Q	Meal-Q	MiniMeal-Q	Meal-Q	MiniMeal-Q
Energy	0.16	0.16	—	—	0.18 (0.01-0.36)	0.18 (0.01-0.33)
Protein	0.22	0.21	0.30	0.31	0.33 (0.17-0.47)	0.34 (0.18-0.48)
Carbohydrates	0.54	0.54	0.62	0.57	0.65 (0.54-0.74)	0.60 (0.48-0.70)
Total fat	0.06	0.02	0.55	0.49	0.57 (0.45-0.67)	0.51 (0.37-0.62)
Saturated fat	0.15	0.11	0.57	0.54	0.60 (0.48-0.70)	0.57 (0.44-0.67)
Monounsaturated fat	0.13	0.08	0.52	0.46	0.56 (0.43-0.67)	0.50 (0.36-0.62)
Polyunsaturated fat	0.23	0.21	0.36	0.35	0.42 (0.25-0.56)	0.40 (0.23-0.54)
Alcohol	0.64	0.65	0.61	0.63	0.73 (0.59-0.82)	0.74 (0.60-0.83)
DLW, energy (CI)^d^	0.42 (0.16-0.68)	0.38 (0.10-0.66)	—	—	—	—

^a^All correlation coefficients were statistically significant (*P* = <.001-.046), except for crude total, saturated and monounsaturated fat for both questionnaires (*P*=.06-.84).

^b^Adjustments for energy were made using the residual method [18].

^c^Deattenuated values were obtained using the formulas suggested by Beaton et al [20] and Liu et al [21]. Confidence intervals were produced using the method suggested by Willett and Rosner [22].

^d^Confidence intervals were obtained using the bootstrap method [23].

**Table 5:** Daily energy and macronutrient intake assessed with the 2 Meal-Q assessments in groups 2 and 3, quartile cross-classifications and crude and energy-adjusted^a^ intraclass correlation coefficients^b^ (ICC) (n=87)^c^.

Energy and macronutrients	Median (IQR) intake			Quartile cross-classifications, %			ICC (95% CI)	
	Meal-Q 1	Meal-Q 2^c^	Difference^d^	Same	Adjacent	Extreme	Crude	Energy-adjusted
Energy (kJ)	7720 (3567)	7383 (3205)	–125 (2497)	51	34	6.9	0.57 (0.42-0.71)	—
Protein (g)	79 (36)	78 (29)	–1.2 (24)	53	40	2.3	0.52 (0.37-0.67)	0.73 (0.63-0.83)
Carbohydrates (g)	209 (122)	206 (113)	0.7 (82)	52	41	2.3	0.64 (0.51-0.76)	0.67 (0.56-0.80)
Total fat (g)	62 (30)	62 (29)	–1.9 (23)	59	26	5.7	0.47 (0.30-0.63)	0.57 (0.43-0.71)
Saturated fat (g)	20 (11)	21 (13)	–0.9 (7.5)	61	25	3.4	0.43 (0.26-0.60)	0.58 (0.44-0.72)
Monounsaturated fat (g)	22 (12)	23 (10)	–0.4 (8.6)	56	32	3.4	0.50 (0.34-0.66)	0.60 (0.46-0.73)
Polyunsaturated fat (g)	13 (9.0)	13 (8.2)	–0.01 (4.84)	57	36	3.4	0.65 (0.53-0.77)	0.68 (0.56-0.79)
Alcohol (g)	4.6 (8.5)	4.3 (7.0)	–1.0 (2.0)	74	22	1.1	0.92 (0.89-0.95)	0.90 (0.87-0.94)

^a^Adjustments for energy were made using the residual method [18].

^b^Intraclass correlation coefficients [24] were computed using 1-way ANOVA with random effects.

^c^Missing values on Meal-Q 2 for 4 participants.

^d^Meal-Q 1–Meal-Q 2; *P*=.27-.96 via Wilcoxon signed rank test.

## Discussion

### Principal Results

This study presents the validity and reproducibility of the new meal- and Web-based interactive Meal-Q, as well as a simulated validation of its shorter version, MiniMeal-Q. Both questionnaires were perceived as easy to use and had a short answering time. Trends of varying bias within the intake range were seen for energy and all macronutrients. Both questionnaires showed good ranking ability for carbohydrates, total fat, saturated fat, monounsaturated fat, and alcohol, whereas energy, protein, and polyunsaturated fat performed less well. Furthermore, Meal-Q showed fair reproducibility.

### Comparison With Prior Work

The Bland-Altman plots of the questionnaires versus WFR and DLW and the plot on WFR versus DLW showed a varying bias within the intake range. Energy and polyunsaturated fat both seemed to be underestimated and overestimated for both questionnaires at higher intake levels. For other macronutrients, the plots indicated that the questionnaires had difficulty assessing higher intakes. In contrast, quartile cross-classification with the WFR showed a fair ranking agreement for most of the nutrients, although a lower agreement was seen for energy and polyunsaturated fat. Similar rankings have been seen in 3 other validation studies of FFQs against food records [25-27] including a Web-based method [25]. In nutritional epidemiology, the association between diet and disease is commonly studied by ranking the dietary intake; therefore, absolute intake is often less important than good ranking order [18]. Hence, despite an underestimation of absolute intake, the ranking agreement for Meal-Q and MiniMeal-Q suggests they are useful in epidemiologic studies regarding most nutrients.

The correlations between the questionnaires and the WFR ranged from 0.18 for energy to 0.74 for alcohol. High correlations between FFQs and diet records are in the order of 0.6-0.7 and it is unlikely that correlations above 0.8 can be obtained [28]. A review of FFQs concluded that the mean correlation with food records of ≥6 days was 0.42 for energy, 0.57 for total fat, 0.53 for protein, 0.58 for carbohydrates, and 0.76 for alcohol [29]. In light of this literature, energy and protein seemed to perform less well, whereas other macronutrients showed correlations within expected ranges. A limitation of the FFQ methodology is the predefined number of food items, frequencies, and portion sizes, which could lead to a “flattened slope” effect in scatter plots [30]. This is a result of respondents consuming little food to unintentionally overreport, and for those consuming a lot to underreport. Correlations from such data would be artificially low. However, a truly small between-person variance would also give similar results [31]. Therefore, the low to moderate correlations seen in this study could reflect a limitation of the questionnaire design, but may also reveal a true small between-person variance. Bland and Altman have discouraged the use of correlation coefficients to evaluate validity because they do not measure agreement [19]. However, because the use of correlations in validation studies is widespread, we have included them to enable comparisons with other studies.

The DLW measurements in group 3 showed that the WFR, Meal-Q, and MiniMeal-Q underestimated energy intake by 17%, 30%, and 36%, respectively. Similar figures for food records and FFQs have been seen in other studies using DLW [32-34]. The Bland-Altman plots showed that the underestimation of energy was considerable for both questionnaires and the large variance indicated difficulties in precision. The underestimation and variance was much smaller for the WFR. Correlations with DLW were moderate for both questionnaires, although the CIs were wide because of the large variance. The correlations were similar to a study by Andersen et al [35], but slightly lower than that of Kroke et al [36]. Despite the underestimation and the large variance, quartile cross-classification with DLW showed a fair ranking agreement, similar to that found by Kroke et al [36].

The moderate to strong quartile cross-classifications of the first and second Meal-Q assessments suggest the questionnaire has fair reproducibility. Correlations between repeated administrations of FFQs in other studies have ranged *r*=0.5-0.8 [31], and Meal-Q showed similar results. The reproducibility might have been affected by the short time period between the Meal-Q assessments, because participants are less likely to have true changes in intake or response after a short compared to a longer period [37]. In addition, it is important to keep in mind that the reproducibility cannot reveal systematic errors, which can be masked in a high correlation between 2 questionnaires.

The high between-person variance captured by MiniMeal-Q as compared to Meal-Q indicates that it is possible to use a shorter questionnaire while still assessing a similar intake range and keeping the ranking ability. Because MiniMeal-Q originates from Meal-Q data and is also compared to the same reference methods, their results become highly related. Therefore, caution must be taken when comparing their assessments and relative validity.

Regular use of the Internet in Sweden is higher among young people compared to older people. Among those aged 16 to 44 years, 88% to 94% use the Internet daily, whereas the proportion among the age groups 45 to 54 years and 65 to 74 years are 82% to 83% and 38% to 49%, respectively [3]. However, access to the Internet is high for all age groups—more than 90% for the young and middle-aged and 67% to 78% for the oldest age group. It is worth noticing that problems with cognition might be an issue in very old age groups, although this would also hold true for dietary assessment using a paper-based questionnaire. Concerns could be raised regarding whether Web-based questionnaires produce different kinds of bias as compared to paper-based questionnaires. However, bias associated with Web-based data collection does not seem to differ from that of paper-based questionnaires as seen in a large Swedish feasibility study of more than 45,000 participants [38].

### Limitations and Strengths

To estimate the validity of a dietary assessment method, 2 statistical assumptions should be fulfilled. First, the assessed dietary intake should be linearly related to true intake. Second, the measurement errors should be independent between the test and the reference method. In this validation study, variables were linearly correlated to the WFR, although energy, protein, and polyunsaturated fat had a weaker linear relationship. The questionnaires rely on memory and have predefined food items, frequencies, and portion sizes, whereas the WFR does not rely on memory, is open-ended, and has direct assessment of portion sizes. Nevertheless, the methods are linked to the same nutrient database and are likewise affected by social desirability, which could lead to an overestimated validity.

The strengths of this validation study include its large sample size and few dropouts. There was also high compliance to the questionnaires, the WFR and DLW. Using the DLW method is an additional strength that enabled an objective estimation of TEE for the evaluation of energy intake. The digital format of the questionnaires and the WFR also substantially reduced the risk of coding errors and missing data. The proportion of underreporters in this study (14/177, 8%) was notably lower compared with some other studies [39-42], even if studies have had proportions in the range of 2% to 85% [43]. The use of individual PAL values from each participant for the Goldberg cut-off is likely to have increased the sensitivity [44] and could be an explanation.

Due to time constraints, the study period had to be kept short. This could have given an overestimation of the validity because the questionnaires and the WFR assessments were performed within a short period of time. Furthermore, the WFR was only performed once. Ideally, several records with independent days spread over a longer time period would have reflected the habitual dietary intake better. However, corrections for within-person variance in the WFR were made to minimize day-to-day variation and energy adjustment was made to avoid variations in intake related to total energy intake. The DLW measurement should also preferably have been done repeatedly over a longer time period to reflect habitual energy intake; however, this was not possible. Furthermore, most of the participants were women and many were students with a nutrition background. Also, the self-selection of participants could have biased the sample in favor of more motivated participants who are more inclined to give accurate answers compared to a sample from the general population [45]. Nevertheless, dietary intake from the WFR was in-line with a Swedish national dietary survey using food records (n=1214) [42], suggesting that the WFR intake would be comparable to an assessment within a more general and less-selected population. Acknowledging the highly educated study sample, the answering time might have been longer in a less-educated population.

### Conclusions

Meal-Q and MiniMeal-Q are 2 Web-based FFQs shown to be highly user-friendly. Despite their short answering time, they had an ability to rank most macronutrient intakes well compared with the reference methods. In addition, Meal-Q showed fair reproducibility.

## Multimedia Appendix 1

Bland-Altman plots showing the differences in energy, protein, carbohydrate, total fat, saturated fat, monounsaturated fat, polyunsaturated fat and alcohol intake assessed with MiniMeal-Q and intake assessed with the WFR plotted against the mean of the two methods (n=163). Macronutrients are energy-adjusted using the residual method [19].

## Abbreviations

BMI: body mass index
DLW: doubly labeled water
FFQ: food frequency questionnaire
ICC: intraclass correlation coefficient
PAL: physical activity level
SLAP: Standard Light Arctic Precipitation
TEE: total energy expenditure
VSMOW: Vienna Standard Mean Ocean Water
VALMA: validation of methods assessing diet and physical activity
WFR: weighed food record
