# Supplementary figures and images for: Two New Meal- and Web-Based Interactive Food Frequency Questionnaires: Validation of Energy and Macronutrient Intake
Source: J Med Internet Res. 2013 Jun 5;15(6):e109. doi: 10.2196/jmir.2458 (PMC3713929; doi:10.2196/jmir.2458)

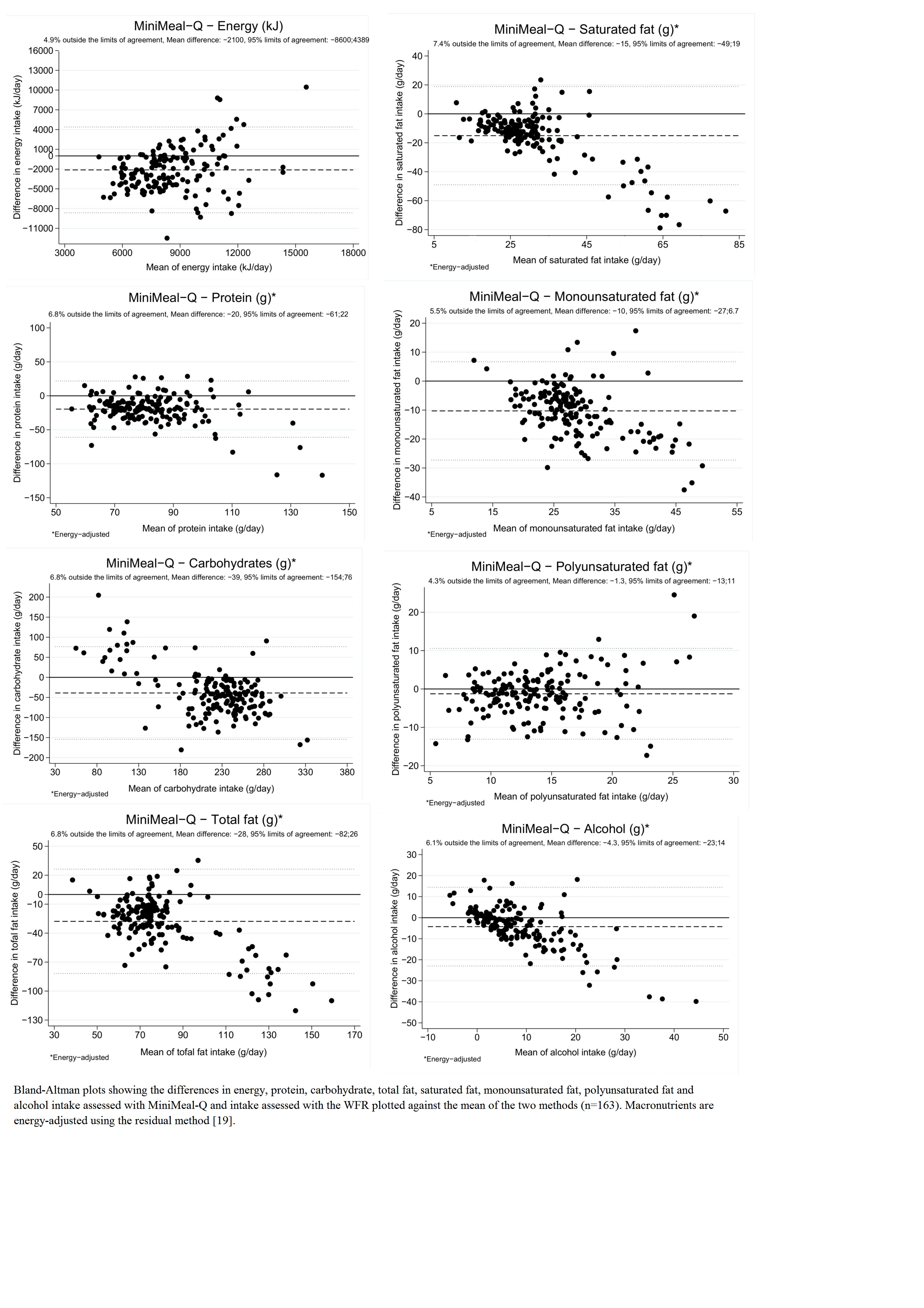

Supplement: Supplementary file 1 [file jmir_v15i6e109_app1.jpg]
